# Sorokiniol: a new enzymes inhibitory metabolite from fungal endophyte *Bipolaris sorokiniana* LK12

**DOI:** 10.1186/s12866-016-0722-7

**Published:** 2016-06-09

**Authors:** Liaqat Ali, Abdul Latif Khan, Javid Hussain, Ahmed Al-Harrasi, Muhammad Waqas, Sang-Mo Kang, Ahmed Al-Rawahi, In-Jung Lee

**Affiliations:** UoN Chair of Oman’s Medicinal Plants and Marine Natural Products, University of Nizwa, Nizwa, 616 Sultanate of Oman; Department of Biological Sciences and Chemistry, College of Arts and Sciences, University of Nizwa, Birkat Al-Mouz, Nizwa, 616 Sultanate of Oman; School of Applied Biosciences, Kyungpook National University, Daegu, 41566 Republic of Korea; Department of Agriculture, Abdul Wali Khan University, Mardan, Pakistan

**Keywords:** Endophytic fungi, Secondary metabolites, *Bipolaris sorokiniana* LK12, Cyclic peptides, Structure elucidation, Enzyme inhibition and antioxidant

## Abstract

**Background:**

Medicinal plants harboring endophytic fungi could carry significant potential for producing bioactive secondary metabolites. Endophytic fungi serve as alternate source of interesting compounds in their natural and modified synthetic forms to treat different diseases. In this regard, endophytic microflora associated with alkaloid-rich medicinal plants *Rhazya stricta* is least known.

**Results:**

We isolated one new bioactive compound sorokiniol (1) along with two known cyclic peptides BZR-cotoxin I (2) and BZR-cotoxin IV (3) from fungal endophyte *Bipolaris sorokiniana* LK12. The structures of the isolated new and known compounds were elucidated through spectroscopic data, including 1D and 2D NMR (^1^H, ^13^C, HSQC, HMBC, and NOESY), mass, and UV. The known peptides (2–3) were characterized by ESI-MS, MS/MS, and by comparing the NMR data with the literature. The isolated metabolites were assayed for their role against enzyme inhibition. Compound 1 was significantly inhibitory towards acetyl cholinestrase while the other compounds (2–3) had moderate anti-lipid peroxidation and urease activities.

**Conclusion:**

The present results suggest that the endophytic microorganism associated with indigenously important medicinal plants can offer a rich source of biologically active chemical constituents which could help in discovering enzyme inhibitory lead drugs.

**Electronic supplementary material:**

The online version of this article (doi:10.1186/s12866-016-0722-7) contains supplementary material, which is available to authorized users.

## Background

Endophytes (bacteria or fungi) reside in the internal tissues of plant at least once in their life cycle. This symbiosis can be oriented towards commensalism or mutualism between host and microbe. In this association, the plant provides a protective sanctuary and accessibility to nutrients, whereas, in return, the endophyte establishes a mutual relationship with host by imparting positive effects by regulating phytohormones and mineral nutrients [1]. The endophytic microorganisms secrete metabolites inside plant’s tissues to improve tolerance against abiotic environmental stresses [2–5]. Various biologically active metabolites are produced by endophytic microorganisms such as alkaloids, steroids, flavonoids, peptides, azaphilones, terpenoids, and phenolics [5–7]. Schulz et al. [1] emphasized on the diversity of active chemical constituents (~51 %) from endophytic microbes in comparison with other soil microflora (~38 %). Currently, there have been many reports of bioactive natural products with significantly higher anticancer, insecticidal, and antimicrobial potentials from fungal endophytes [8].

One of the interesting examples is the production of paclitaxel (Taxol – an anticancer drug) from endophyte *Pestalotiopsis microspora* [9]. Such potential has increased the interest to search more numbers of novel metabolites from fungal endophytes [3, 10]. In present work, *Rhazya stricta* was selected on the basis of its indigenous medicinal importance whilst to explore associated endophytic fungus and their ability to produce biologically active chemical constituents. *Rhazya stricta* wildly grows in various countries of Middle Eastern regions and South Asia. It is medicinally important plant and is famous for alkaloids contents [11]. Approximately, 100 different kinds of alkaloids are isolated from *Rhazya stricta* [12, 13]. Some of the chemical compounds isolated from this plant have been identified to possess strong potential in various pharmacological properties [13].

*Rhazya stricta*, being rich in wide array of chemical constituents, was selected with the aim to assess the symbiotic endophytic fungi and potential metabolites. The endophytic wealth has not been efficiently explored from *Rhazya stricta*. In a recent report we isolated fungal endophyte *Bipolaris sorokiniana* LK12 from the leaf parts of *R. stricta* [14]. This strain was characterized for the production of bipolarisenol – a new metabolite which is a derivative of radicinol. The metabolite was found to significantly inhibit the activities of urease and acetyl cholinesterase enzymes [14].

**Fig. 1:**
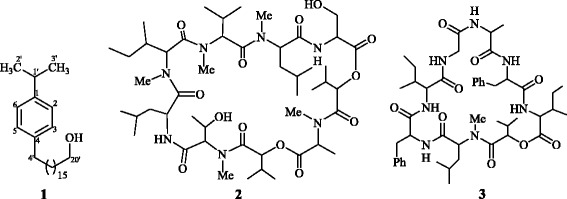
Structures of the isolated compounds (1–3) from fungal endophyte *Bipolaris sorokiniana* LK12

Looking at its metabolomics potential and bioactivities, we further continued the exploration of bioactive chemical compounds from this endophytic fungal strain. In this regards, current study was aimed to isolate and characterize biologically active chemical constituents produced from *B. sorokiniana* LK12 through advanced chromatographic and nuclear magnetic resonance spectroscopy techniques. The results revealed that a new secondary metabolite, sorokiniol (1), along with two known cyclic depsipeptides, BZR-cotoxin I (2) and BZR-cotoxin IV (3) (Fig. 1), have been isolated whilst the chemical structures were elaborated through advanced spectral techniques. To understand any biological role of isolated metabolites, the compounds were assessed for their enzyme inhibition activities.

## Methods

### Isolation, growth and molecular identification of endophytic fungi

The fungal endophyte *B. sorokiniana* LK12, isolated from the leaf parts of *Rhazya stricta* was identified and grown as described in detail by Arnold et al. [15] and by Khan et al. [14]. Briefly, the isolated fungus (RSL-1.2) was grown in seven liters of Czapek broth comprising peptone (1 %), glucose (1 %), KCl (0.05 %), FeSO_4_.7H_2_O (0.001 %), MgSO_4_.7H_2_O (0.05 %), streptomycin (80 ppm) and pH 7.5 ± 0.2. The cultured fungus inoculated in liquid broth was grown on shaking incubator for twenty one days at 28 °C. The broth was centrifuged (5,000*x*g at 4 °C for 10 min) to separate the culture filtrate (CF) and mycelia (120.4 g). Approximately, one gram of fungal cells were lyophilized for DNA extraction, PCR analysis, and sequencing for identification.

The fungal DNA was extracted as described Khan et al. [14]. The fungus was identified through sequencing internal transcribed spacer (ITS) regions. A set of universal primers ITS-1 (5’-TCCGTAGGTGAACCTGCGG-3’) and ITS-4 (5’-TCCTCCGCTTATTGATATGC-3’) was also used to amplify through PCR. The PCR product was sequenced by Macrogen Inc. (Seoul, South Korea). The sequence obtained was BLASTn searched (NCBI) to compare the sequence homology of KL12 nucleotides and with that of related fungi using ITS regions. The homologous sequences were aligned using Clustal W (1.6) in MEGA 6.0 [16, 17]. A maximum parsimony tree was constructed. Bootstrap replications (1000) were used for nodes in phylogenetic tree. The sequence was submitted to NCBI GenBank for the accession number.

### Extraction and purification of compounds

The mycelial cells (120.0 g) and culture filtrate (seven liters) were partitioned with equal amount of ethyl acetate at least three times. Both extracts were dried *in vacuo* and traces of water was removed by anhydrous sodium sulfate. The dried extract was further concentrated under reduced pressure to get 2.1 g of crude EtOAc extract. The silica gel column chromatography was performed on the ethyl acetate (EtOAc) extract by using various polarity (*n*-hexane/ EtOAc) solvent system, which afforded 5 fractions; A to E. The fifth fraction (E) was further purified through a fully preparative recycling HPLC (High Performance Liquid Chromatography; JAI, Japan). A continuous flow rate of 3.5 mL/min (6:4 EtOAC/*n*-hexane) was maintained and compound 1 (5.1 mg) was obtained at 23 min retention time with five recycles.

### Chromatography and spectroscopic techniques for identification

The measurement of optical rotations was performed on a polarimeter (JASCO DIP 360). The IR spectra were obtained through ATR-Tensor 37 spectrophotometer by Bruker. To record the ESI mass spectra the QSTAR XL mass spectrometer by Applied Biosystems was used with a capillary voltage set from 5–5.5 kV. The NMR spectra (^1^H and ^13^C) were recorded on Bruker spectrometer operating at 600 MHz (150 MHz for ^13^C). The *δ* values on the chemical shift scale are reported in ppm, while the J values of the coupling constants are given in Hz. Purification of minor compounds was performed by recycling HPLC by using a 7:3 EtOAc/*n*-hexane solvent system in a silica gel column with a flow rate of 4 mL/min. Pre-coated aluminium silica gel sheets (60 F-254, Merck) were used for TLC (thin layer chromatography), which were visualized under UV light at 254 and 366 nm. Ceric sulphate and ninhydrin reagent were used for spraying the TLC followed by heating.

### Identification of the metabolites

The isolated metabolites were characterized by spectroscopic data (UV, IR, ^1^H-NMR, ^13^C-NMR, 2D NMR, ESI and MS/MS studies). Compound 1 was characterized as a new constituent, whereas compound 2 and 3 were identified as earlier reported BZR-cotoxin I and BZR-cotoxin IV respectively. HR-EI-MS data showed that the molecular weight of chemical compound 1 was 374.3543 (calcd. for C_26_H_46_O 374.3549), whereas ESI-MS indicated the molecular weight of compound 2 as 968.6287 [M + H]^+^ (calcd. for C_48_H_86_N_7_O_13_, 968.6283) and compound 3 as 876.5239 [M + H]^+^ (calcd. for C_47_H_70_N_7_O_9_, 876.5244).

Compound 1: Pale yellow gummy solid (24.5 mg); m.p. 170–175 ° C, [α]_D_^30^ +0.8° (CHCl_3_; c 0.0013,); IR υ_max_ (CHCl_3_) 3400 (OH); 2922, 1591, and 1463 (aromatic functionalities); ^1^H-NMR (600 MHz, CDCl_3_): δ 7.21 (2H, d, *J* = 8.4 Hz), 6.94 (2H, d, *J* = 8.4 Hz), 3.67 (2H, m), 2.83 (1H, m), 2.27 (2H, m), 1.53-1.21 (16H, overlap signal), 1.29 (14H, s), 1.23 (6H, d, *J* = 6.1 Hz); ^13^C-NMR (150 MHz, CDCl_3_): δ 148.7 (C-1), 139.2 (C-4), 126.9 (C-2, C-6), 117.2 (C-3, C-5), 56.9 (C-20′), 36.9 (C-4′), 32.4 (C-5′), 31.9 (C-1′), 30.9 (C-19′), 29.2 (C-6′ to C-18′), 22.7 (C-2′, C-3′); EI-MS (*m/z*): 374 [M]^+^, 119 [M – side chain alcohol]^+^; HR-EI-MS (*m/z*): 374.3543 (calcd. for C_26_H_46_O 374.3549).

Compound 2: Colorless amorphous powder (4.9 mg); IR υ_max_ (CHCl_3_) 3430 (OH); 2950, 2890, 1755, 1670, and 1645; ^1^H-NMR (600 MHz, CDCl_3_): δ 8.11 (1H, d, *J* = 8.2 Hz, NH), 7.28 (1H, br s, NH), 5.52 (1H, d, *J* = 10.1 Hz), 5.43 (1H, d, *J* = 6.7 Hz), 5.14 (1H, d, *J* = 10.7 Hz), 5.06 (1H, d, *J* = 10.4 Hz), 5.02 (1H, d, *J* = 8.5 Hz), 4.86 (2H, m), 4.71 (1H, m), 4.64 (1H, m), 4.23 (1H, br s), 3.80 (1H, br s), 3.57 (1H, dd, *J* = 9.8, 5.8 Hz), 3.27 (3H, s), 3.12 (3H, s), 2.93 (3H, s), 2.85 (3H, s), 1.39 (3H, d, *J* = 6.7 Hz), 1.25 (3H, d, *J* = 6.4 Hz), 2.45-1.20 (11H, m), 1.07 (3H, d, *J* = 6.7 Hz), 1.20-0.86 (28H, m), 0.81 (3H, d, *J* = 6.4 Hz), 0.73 (3H, d, *J* = 6.7 Hz); HR-ESI-MS (*m/z*): 968.6287 [M + H]^+^ (calcd. for C_48_H_86_N_7_O_13_, 968.6283).

Compound 3: Amorphous powder (5.3 mg); IR υ_max_ (CHCl_3_) 3430, 2975, 2880, 1730, 1680, 1525; ^1^H-NMR (600 MHz, CDCl_3_): δ 7.54 (1H, br s, NH), 7.38 (1H, d, *J* = 7.3Hz, NH), 7.35-7.17 (11H, m), 7.06 (1H, d, *J* = 5.6 Hz, NH), 7.00 (1H, br s, NH), 6.69 (1H, d, *J* = 8.9 Hz, NH), 4.85 (1H, d, *J* = 5.8 Hz), 4.72 (1H, m), 4.54 (1H, t, *J* = 7.8 Hz), 4.34 (1H, dd, *J* = 7.0, 7.3 Hz), 4.05-3.85 (2H, m), 3.78 (1H, dd, *J* = 4.3, 16.3 Hz), 3.68 (1H, m), 3.49-3.30 (3H, m), 3.24-3.07 (2H, m), 3.02 (3H, s), 2.20-1.10 (10H, m), 1.03 (3H, d, *J* = 7.0 Hz), 0.99 (3H, d, *J* = 6.7 Hz), 0.90-0.85 (12H, m), 0.79 (3H, t, *J* = 7.3 Hz); HR-ESI-MS (*m/z*): 876.5239 [M + H]^+^ (calcd. for C_47_H_70_N_7_O_9_, 876.5244).

### Enzyme inhibition assays and anti-lipid peroxidation

The ethyl acetate extract and the three compounds were subjected for antioxidant and enzyme suppression assay. The inhibition potential of isolated compounds was evaluated by modified thio-barbituric acid reactive substances (TBARS) method [18]. The oxidation of liposome (phosphatidyl-choline, Sigma – Germany; 50 mg ml^−1^) was caused by a mixture (0.200 ml) of iron chloride (FeCl_3_, 1 mM) and potassium chloride (KCl, 300 mM) in the presence of the sample/compound (0.050 ml). Ascorbate (0.16 mM; 0.125 ml) was used to initiate the peroxidation. The reaction mixture was incubated at 37 °C for 20 min. Mixture of TBA (0.38 %) and TCA (0.750 ml 1.5:1 (v/v) solution) was then added to the reaction mixture, and kept on 95 °C for half an hour. The appearance of pink color was read on ELISA microplate reader (xMark, BioRad, USA) at 535 nm. A control without test sample was used and the experiment was repeated three times. The percent inhibition was measured using formula: (1- At/Ao) x 100; where At and Ao are the absorbance for sample, blank and standard after 30 min incubation.

The method of Golbabaei et al. [19] was used to assess the urease enzyme (EC 3.5.1.5) inhibition activities. Briefly, a 0.055 ml urea (100 mM) in phosphate buffer (0.01 M LiCl_2_, 1.0 mM EDTA, 0.01 M K_2_HPO_4_.3H_2_O, pH 8.2), 0.025 ml Jack bean Urease (2 units/mL; Sigma, Germany), and various concentrations of the chemical compounds (10–100 μg/mL) were combined in the reaction mixture with incubation for 15 min in a 96-well plate at 37 °C. The indophenol method was used to evaluate the urease inhibitory activity by measuring the ammonia production. The alkali reagent (0.1 % NaOCl, 0.070 ml and 0.5 % sodium hydroxide) and the phenol reagent (0.045 ml, 1 % w/v phenol and 0.005 % w/v sodium nitroprusside) were mixed in each well and the change in absorbance per minute was measured after 50 min at 630 nm, by using ELISA microplate reader. All the experiments were repeated thrice and thiourea (10–200 μg/mL) was used as the standard inhibitor (95 ± 1.50 % Inhibition). The percentage inhibition was calculated by = 100 – (OD_test_ /OD_control_) × 100.

Acetyl Cholinestrase Enzyme (AChE) inhibition was measured by using a marginal modification in Ingkaninan et al. method [20]. In this method the substrate acetylthiocholine iodide (ATCI, 15 mM) is hydrolysed enzymatically to produce thiocholine, which in turn reacts with Ellman’s reagent (DTNB, 3 mM), which is measured at 412 nm in ELISA microplate reader. AChE enzyme used in the activity was obtained from electric eel (844 U/mg protein). The stock solutions of enzyme were kept at −80 °C. Tris–HCl buffer (0.02 M MgCl_2_ and 0.1 M NaCl) was used to dissolve DTNB, whereas ATCI solution was made in deionized water.

A mixture of 20 μl of 0.25 U/ml of AChE, 40 μl of buffer (50 mM Tris pH 8.0), and 100 μl of DTNB were added along with 20 μl of different concentrations of compounds to each well in 96-well plates. The plate was incubated at 25 °C for 15 min. The absorbance of the reaction mixture was read at 412 nm in ELISA spectrophotometer (xMark BioRad, USA). The negative control did not had sample in the assay mixture. To start the reaction, 20 μl of ATCI was added to assay mixture and the rate of hydrolysis in acetylthiocholine read every 2 min for 20 min. All the reactions were performed thrice and Galanthamine was used as the positive control and similar percentage inhibition formula was used mentioned earlier for urease enzyme.

### Statistical analysis

The values shown are the mean of three replicates. Linear regression analysis were performed with p values ranged to 0.05. Graphical representations were done by using GraphPad Prism software 6.01 package (GraphPad Software, Inc., CA, USA).

## Results and discussion

### Endophyte identification and screening bioactivity

**Fig. 2:**
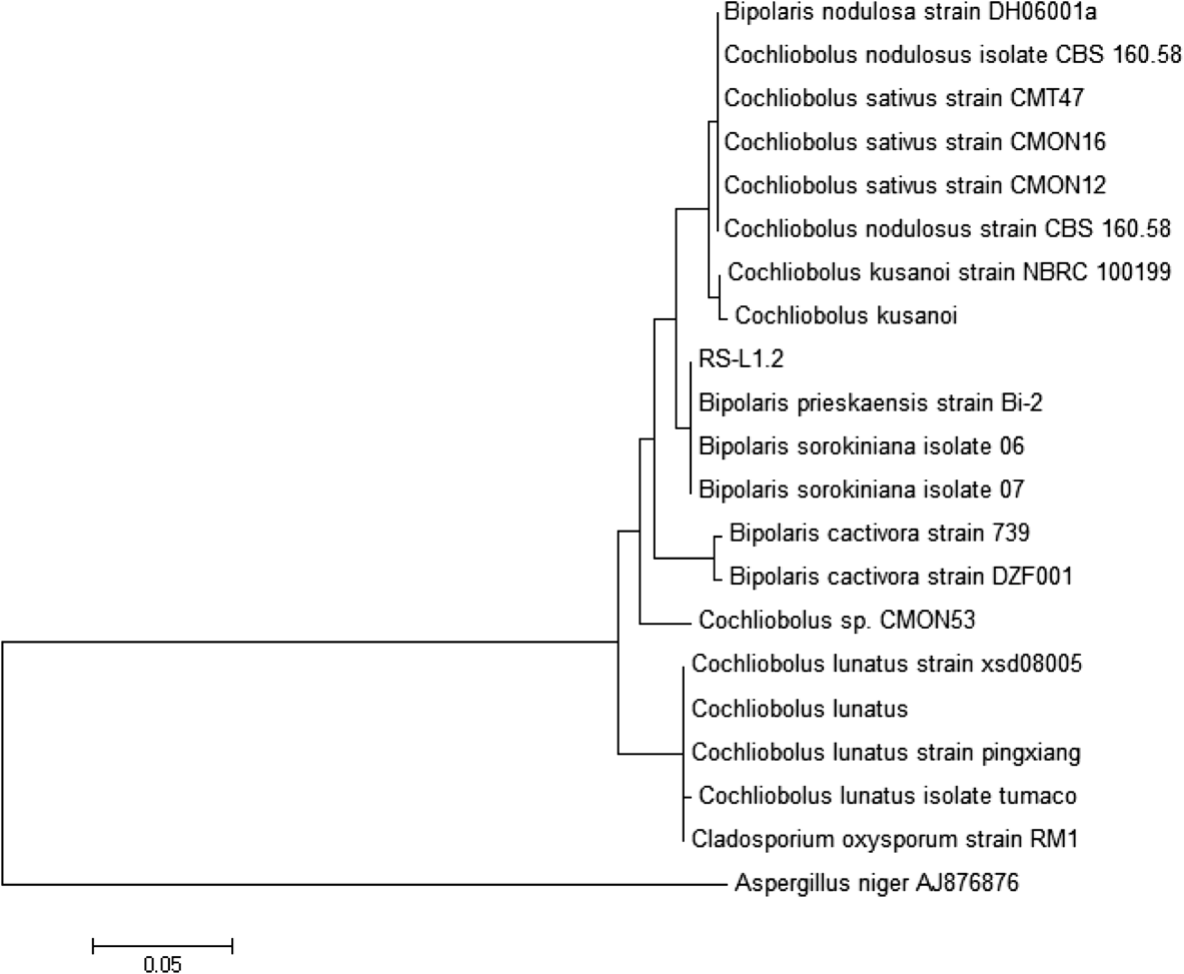
Phylogenetic analysis of the ITS rDNA sequence of RSL-1.2 isolated from leaves of *R. stricta*. The analysis showed 99 % sequence homologies with *Bipolaris sorokiniana. Aspergillus niger* was used as an out-group

Endophytic fungus RSL-1.2 was identified by gDNA extraction, PCR sequencing and amplification of ITS regions. The phylogenetic analysis of RSL-1.2 was carried out by using MEGA 6.0. With the help of neighbor joining (NJ) and maximum parsimony (MP) methods a consensus cladogram was established using 20 (19 references and 1 clone) homologues ITS sequences. During BLASTn search, RSL-1.2 represented the highest sequence homology, query coverage, and lowest E values with *B. sorokiniana*. In dendrogram, RSL-1.2 formed a 99 % bootstrap level of support with *B. sorokiniana* (Fig. 2). The aligned ITS sequence was deposited in the NCBI GenBank for an accession no. KP715347 as *B. sorokiniana* LK12 strain.

The EtOAc extract of the fungus *B. sorokiniana* LK12 was assessed for its antioxidant capacity using anti-lipid peroxidation, ABTS and superoxide anion radical scavenging activities. The results shown that the extract exhibited strong radical (superoxide anion and ABTS) scavenging potential in comparison to control (Additional file 1). The anti-lipid peroxidation activity was moderate as compared to the control.

### Isolation and structural elucidation of secondary metabolites

Looking at the bioactive potentials of the EtOAc extract of endophyte, it was subjected to chromatographic studies to isolate bioactive secondary metabolites which yielded three compounds (1–3). Compound 1 was obtained in the form of pale yellow gummy solid by repeated chromatography experiments while using methanol/ethyl acetate (5 %) in a silica gel column. The molecular formula was established as C_26_H_46_O on the basis of EI-MS (M^+^ at *m/z* 374), along with the information obtained through ^13^C-NMR spectra of compound 1 (Additional file 2). The molecular formula C_26_H_46_O was further confirmed through high resolution mass spectrum (HR-MS), which showed the molecular ion peak of 1 at *m/z* 374.3543 (calcd. for C_26_H_46_O 374.3549). The ^1^H-NMR spectrum of compound 1 exhibited two signals for two protons each at δ 6.94 (d, *J* = 8.4 Hz, 2H) and 7.21 (d, *J* = 8.4 Hz, 2H). These coupling constants along with the multiplicities of the aromatic protons indicated the *para*-substitution of benzene ring in compound 1. An upfield doublet for six protons at δ 1.23 (d, *J* = 6.1 Hz, 6H) and a multiplet for one proton at δ 2.83 (m, 1H) were indicative of the presence of isopropyl substituent at benzene ring. The second substituent was in the form of a long chain alcohol, which was confirmed by the combined MS and NMR spectral data. The down field multiplet at δ 3.67 (m, 2H) was assigned to the methylene moiety directly attached to hydroxyl group, whereas the signal appeared at δ 2.27 (m, 2H) was assigned to the methylene group adjacent to benzene ring. The other methylene groups in the chain appeared between δ 1.53 and 1.21 including a singlet at δ 1.29 (s, 14H). The length of the chain was further confirmed by the fragments appearing in EI-MS at *m/z* 31, 45, 59, 73, 87, 101, 115, 129, 143, 157, 171, 185, 199, 213, 227, 241, and 255. Similarly, the presence of isopropyl group and the *para*-substituted benzene ring was confirmed through fragments at *m/z* 43, 76, and 119.

**Fig. 3:**
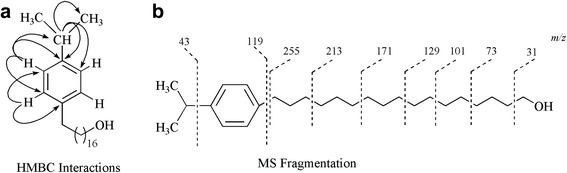
Key HMBC Interactions (**a**) and Major Mass Fragmentations (**b**) in Compound 1

^13^C-NMR spectra of compound 1 (BB, DEPT) indicated the presence of two signals for two sp^2^ carbons each at δ 117.2, 126.9, two quaternary carbons at δ 139.2 and 148.7, an upfield signal for two methyls at δ 22.7, a methine signal at δ 31.9, and signals for seventeen methylene, including a downfield signal at δ 56.9, thus supporting the *para*-substituted benzene ring with isopropyl group and an alcoholic long chain substituent. These assignments were further confirmed by HMBC interactions (Fig. 3) and the structure was finally confirmed as sorokiniol (1), after the producing organism *B. sorokiniana*. Biosynthetically, compound 1 is similar in structure to urushiol class of compounds belonging to the plants of anacardiaceae family in the genus toxicodendron [21]. The biosynthesis of urushiols involves the decarboxylation and hydroxylation reactions of anacardic acid which is the product of extension of three malonyl-CoA units with palmitoleoyl-CoA followed by reduction and aldol cyclization [22].

**Fig. 4:**
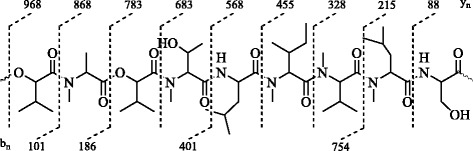
Fragmentation Pattern of Protonated BZR-cotoxin I (2)

Compound 2 was isolated from the crude extract through repeated column chromatography in semi-pure form. Further purification was done by recycling HPLC using 7:3 EtOAc/n-hexane with a flow rate of 4 ml/min after three recycles at an elution time of 21.3 min. Compound 2 was isolated in semi-pure form at a range of 65 % to 75 % ethyl acetate/*n*-hexane system from the crude extract through repeated column chromatography. Further purification was done by recycling HPLC using 7:3 ethyl acetate/*n*-hexane with a flow rate of 4 ml/min after three recycles at an elution time of 21.3 min. The structure of compound 2 was elucidated by the combined spectroscopic analyses including ESI-MS and NMR spectroscopy. The molecular formula of secondary metabolite 2 was established as C_48_H_85_N_7_O_13_ by HR-ESI-MS on the basis of *pseudo*-molecular ion peak at *m/z* 968.6287 [M + H]^+^ (calcd. for C_48_H_86_N_7_O_13_, 968.6283). Moreover, the major linear peptide (Fig. 4) was produced by the cleavage of ester bond between serine amino acid and 2-hydroxy-3-methylbutanoic acid. This linear peptide produced a main series of adjacent *y*_*n*_ peaks at *m/z* 868, 783, 683, 568, 455, 328, 215, and 88 corresponding to the successive losses of 2-hydroxy-3-methylbutanoic acid, *N*-methylalanine, 2-hydroxy-3-methylbutanoic acid, *N*-methylthreonine, leucine, *N*-methylisoleucine, *N*-methylvaline, and *N*-methylleucine, respectively. Similarly, *b*_*n*_ ions were detected at *m/z* 101, 186, 401, and 754.

All these results suggested the sequence of amino acids for the linearized peptide ion derived from BZR-cotoxin I and thus confirmed the structure 2 for the cyclic depsipeptide. Compound 2 showed two amide protons and five downfield methyl signals in ^1^H-NMR spectrum, whereas, nine signals for carbonyl groups were observed in ^13^C-NMR spectrum. The sequence of peptides in the molecule was further confirmed by the HMBC analysis.

The ^13^C-NMR of 2 showed 48 carbon signals which was in correlation with molecular formula, C_48_H_85_N_7_O_13_. The DEPT experiment revealed the multiplicities while HMQC experiments suggested the attachments of the assigned protons. Specific resonances for peptides were observed in ^1^H-NMR spectrum of compound 2 in the form of broad singlets at δ 7.97 and 7.33 ppm, which were assigned to amide NH protons. The peptide α protons appeared in the form of doublet of doublet and in some cases in the form of multiplets between δ 3.19 and 4.79 ppm. The upfield signals in the region δ 0.81 to 1.27 were assigned to the methyl groups of amino acids whereas the methyl groups connected with amide nitrogen appeared between δ 2.81 and 3.36. Furthermore, the purified product had nearly identical physical and spectral properties as those of the reported cotoxin-I, which was previously reported to be produced by *Bipolaris zeicola* race 3 [23].

**Fig. 5:**
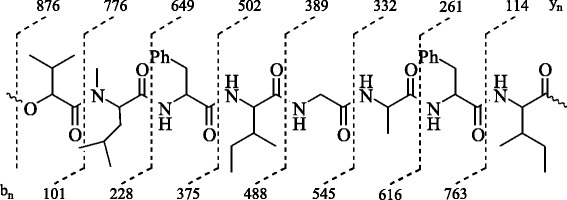
Fragmentation Pattern of Protonated BZR-cotoxin IV (3)

Compound 3 was also isolated in semi-pure form from the crude extract through repeated column chromatography at 80 % to 90 % ethyl acetate/*n*-hexane system. The final purification was obtained through recycling HPLC using 7:3 EtOAc/*n*-hexane with a flow rate of 4 ml/min after three recycles at an elution time of 25.4 min. The structure of compound 3 was elucidated by combined ESI-MS and NMR spectral analysis. Pseudo molecular ion peak of the protonated molecule [M + H]^+^ was observed at *m/z* 876.5239 (calcd. for C_47_H_70_N_7_O_9_, 876.5244) which was helpful to determine the molecular formula C_47_H_69_N_7_O_9_ for compound 3. The major linear peptide (Fig. 5) was produced by the cleavage of ester bond between isoleucine amino acid and 2-hydroxy-3-methylbutanoic acid. This linear peptide produced a main series of adjacent *y*_*n*_ peaks at *m/z* 776, 649, 502, 389, 332, 261, and 114 corresponding to the successive losses of 2-hydroxy-3-methylbutanoic acid, *N*-methylleucine, phenylalanine, isoleucine, glycine, alanine, and phenylalanine, respectively. Similarly, *b*_*n*_ ions were detected at *m/z* 101, 228, 375, 488, 545, 616, and 763.

All these results suggested the sequence of amino acids for the linearized peptide ion derived from BZR-cotoxin IV and thus confirmed the structure 3 for the cyclic depsipeptide. The ^1^H-NMR spectrum of 3 showed six amide protons and one downfield methyl singlet. Characteristic peptide resonances included broad singlets at δ 7.31 and 7.89 ppm, which were assigned to amide NH protons. Additional signals in the aromatic region were assigned to the benzene ring of phenylalanine amino acids. The peptide α protons appeared in the form doublet of doublet and in some cases in the form of multiplets between 3.92 and 4.79 ppm.

In correlation with the molecular formula C_47_H_69_N_7_O_9_, the ^13^C-NMR spectrum of compound 3 contained resonances for 47 carbons including eight signals for the carbonyl groups in the downfield region. The DEPT analysis suggested the multiplicities of carbon resonances while HMQC analysis showed the assignments of attached protons. The sequence of amino acids in the peptide was further confirmed through HMBC experiments. Based on the above discussion and the comparison of the physical and spectral data with those reported in literature, compound 3 was thus confirmed as cotoxin-IV, which was earlier reported to be produced by *Bipolaris zeicola* race 3 [24].

### Enzyme inhibitive potential of secondary metabolites

**Fig. 6:**
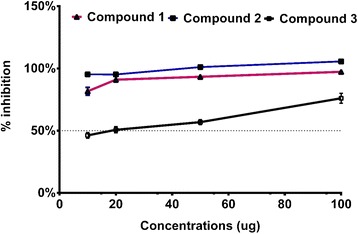
Enzyme Acetyl Cholinesterase inhibition activities of compound 1 – 3. The graphical lines shows standard error of means of three replications

**Fig. 7:**
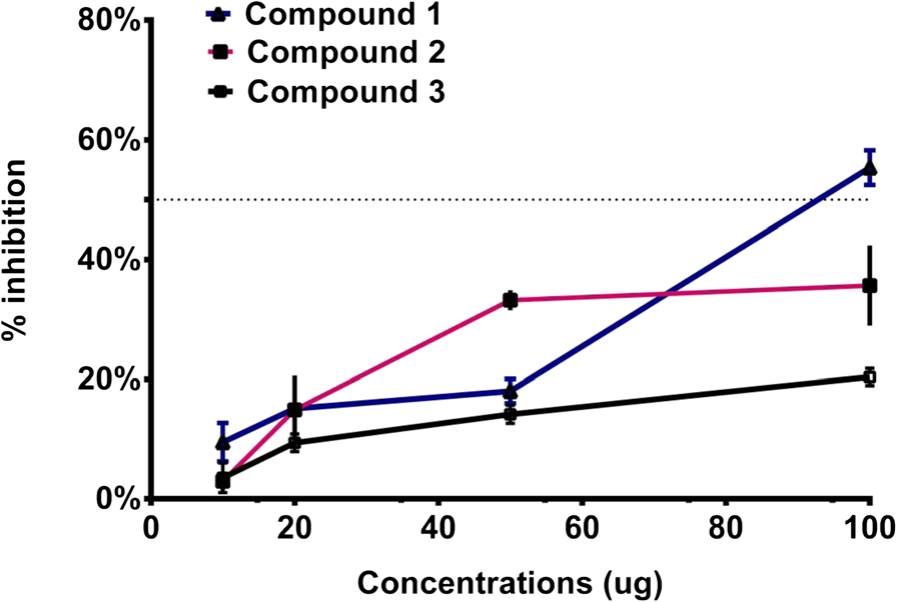
Enzyme Urease inhibition activities of compound 1 – 3. The graphical lines shows standard error of means of three replications

**Fig. 8:**
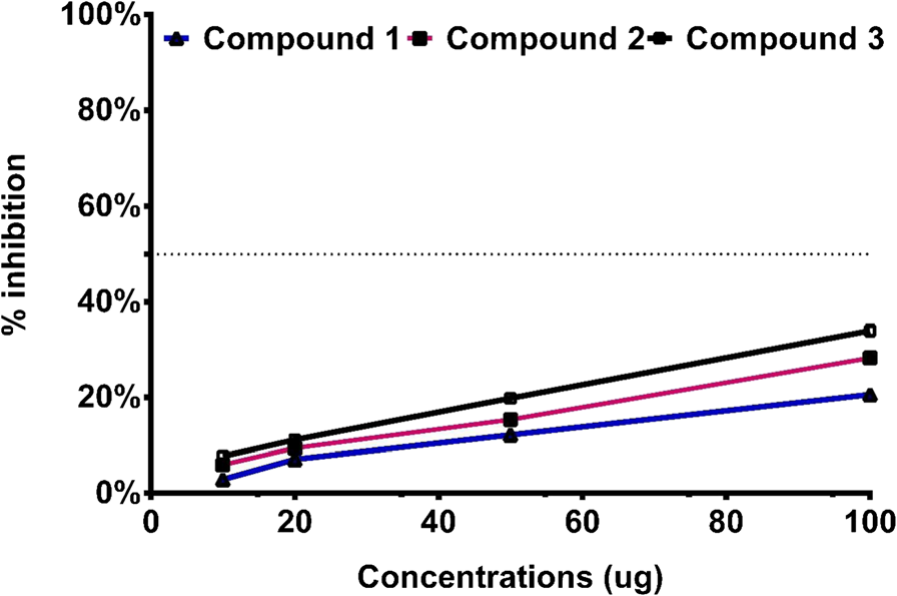
Anti-Lipid Peroxidation activities of compound 1 – 3. The graphical lines shows standard error of means of three replications

The purified compounds 1–3 were subjected to bio-assays against acetyl cholinesterase (AChE) and urease enzyme inhibition whilst these were also assessed for their anti-lipid peroxidation potential. Using a concentration gradient, compound 1 and 2 exhibited significantly higher AChE enzyme inhibition as compared to compound 3 (Fig. 6). The EC_50_ activity for compound 1 was 3.402 + 0.08 μg/mL (R^2^ = 0.89) using linear regression curve fitting with EC_50_ Shift in Graphpad Prism. Compound 3 showed a dose-dependent response against AChE enzyme inhibition. In case of urease enzyme inhibition, only compound 1 showed 50 % inhibition at maximum concentration whilst other compounds (2 and 3) did not suppressed the enzyme activity (Fig. 7). A similar pattern of low level of anti-lipid peroxidation activity was shown by compound 3 (Fig. 8). The current results conclude that the three compounds exhibit significant AChE enzyme inhibition pattern.

Previously, there was no report of enzyme inhibition activity of bioactive metabolites isolated from *B. sorokiniana* LK12, however, a wide majority of fungal endophytes from various plants have been reported to play a vital role against suppression of enzymatic disorders. Antioxidant activities are frequently investigated for endophytes (e.g., *Botryosphaeria dothidea* and *Aspergillus awamori*) isolated from medicinal plants (*Melia azedarach* L. and *Rauwolfia serpentina* Benth, respectively) [8, 25]. Liu et al. [26] isolated *Xylaria* sp. from the stem of ancient medicinal plant *Ginkgo biloba* and studied the potential of this species to produce antioxidants, where the organism exhibited a strong radical scavenging activity. Similarly, Huang et al. [27] investigated the total antioxidant and total phenolic contents of a repository of 292 endophytes from 29 traditional Chinese medicinal plants using improved ABTS methods. Similarly, Rodrigues et al. [28] and Xiao et al. [29] suggested various endophytic fungi from different host plants can show potent source of essential enzyme inhibition activities such as AChE inhibition.

On the other hand, alzheimer’s disease, for which AChE inhibition emerged as the major therapeutic target, only approved drug is galantamine [28]. We used this to compare our results, which were very encouraging; however, further studies at molecular level would be needed to get more insight of receptors involved in the inhibition of AChE [29]. The present results also suggest that the endophytic microorganism of biologically active medicinal plants also carries interesting bioactivities which could help in discovering lead drugs.

## Conclusion

The present results showed that fungal endophyte associated with medicinal plants can possess a potent ability to secrete biologically active metabolites. In current case, *B. sorokiniana* LK12 produced a new compound, sorokiniol along with two known cyclic peptides BZR-cotoxin I and BZR-cotoxin IV. The chemical constituents exhibited significantly inhibitory effects towards acetyl cholinestrase while moderate urease and anti-lipid peroxidation activities. Further studies at broader enzyme kinetics level could further help in understanding the enzyme inhibitory role of chemical constituents isolated from endophytes.

## Abbreviations

AChE, acetyl cholinestrase; DDW, deionized distilled water; HPLC, recycling preparative High Performance Liquid Chromatography; ITS, culture medium (culture filtrate–CF), internal transcribed regions; MP, maximum parsimony; NJ, neighbor joining; PDA, potato dextrose agar; TBARS, thiobarbituric acid reactive substances; TLC, thin layer chromatography

## Additional files

Additional file 1: Figure S1. Antioxidant activity of the ethyl acetate extract (1.0 mg/mL) of the *B. sorokiniana LK12*. ABTS, LPO – anti-lipid peroxidation; O_2_- superoxide anion. Bars shows standard error of five replicates of each activity. For positive control, ascorbic acid was use. (DOC 824 kb) Additional file 2: Figure S2. Nuclear Magnetic Resonance spectroscopic analysis of compound 1-3. (PDF 333 kb)
