# Supplementary material for: Sorokiniol: a new enzymes inhibitory metabolite from fungal endophyte Bipolaris sorokiniana LK12
Source: BMC Microbiol. 2016 Jun 9;16:103. doi: 10.1186/s12866-016-0722-7 (PMC4899901; doi:10.1186/s12866-016-0722-7)
Supplement: Additional file 1: Figure S1. — Antioxidant activity of the ethyl acetate extract (1.0 mg/mL) of the B. sorokiniana LK12. ABTS, LPO – anti-lipid peroxidation; O2- superoxide anion. Bars shows standard error of five replicates of each activity. For positive control, ascorbic acid was use. (DOC 824 kb) [file 12866_2016_722_MOESM1_ESM.doc]

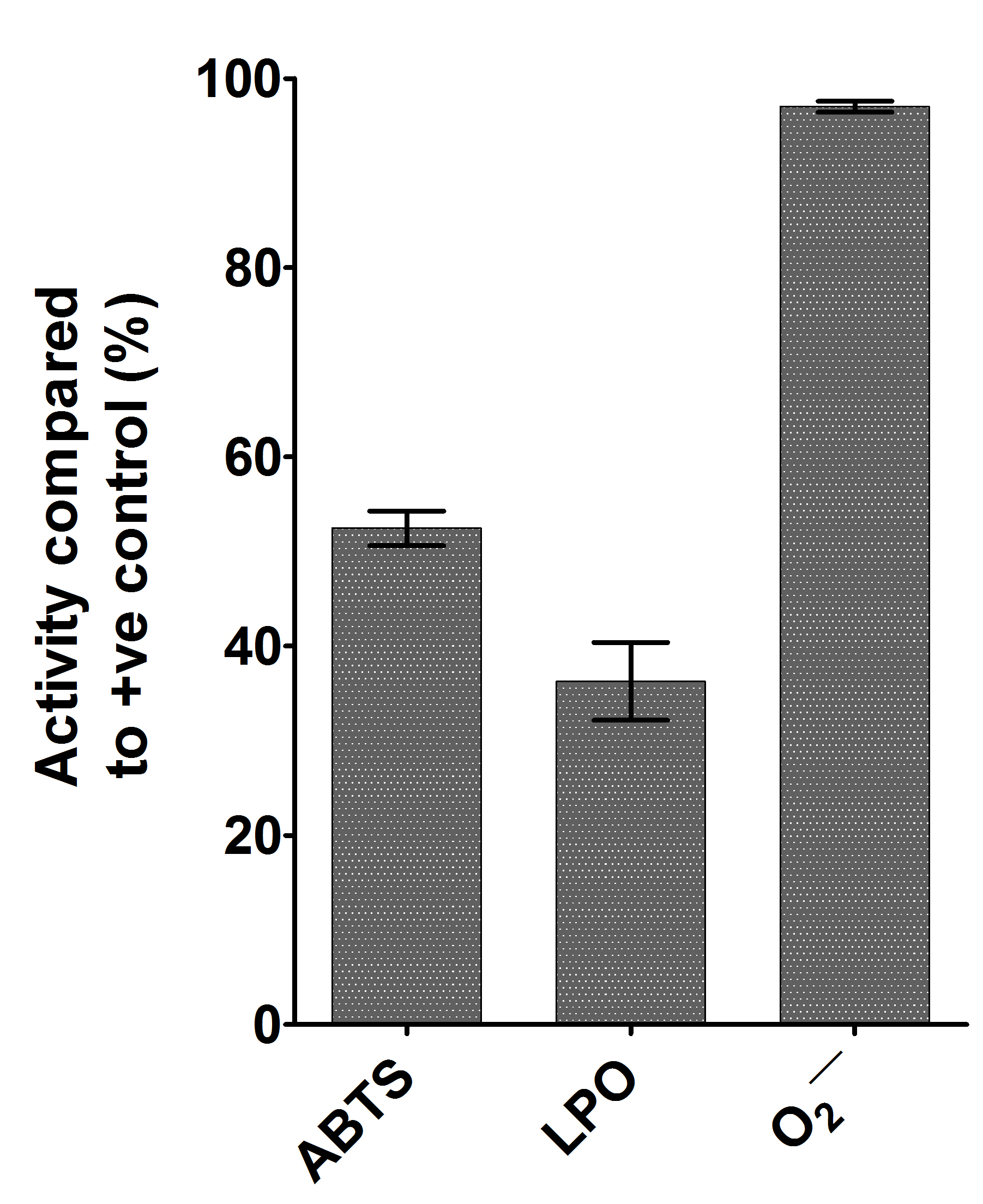


**Supplementary Figure 1**. Antioxidant activity of the ethyl acetate extract (1.0 mg/mL) of the *B. sorokiniana LK12*. ABTS, LPO – anti-lipid peroxidation; O_2_- superoxide anion. Bars shows standard error of five replicates of each activity. For positive control, ascorbic acid was use.
