# Supplementary material for: Sorokiniol: a new enzymes inhibitory metabolite from fungal endophyte Bipolaris sorokiniana LK12
Source: BMC Microbiol. 2016 Jun 9;16:103. doi: 10.1186/s12866-016-0722-7 (PMC4899901; doi:10.1186/s12866-016-0722-7)

^{1}H NMR Spectrum of Compound **1**

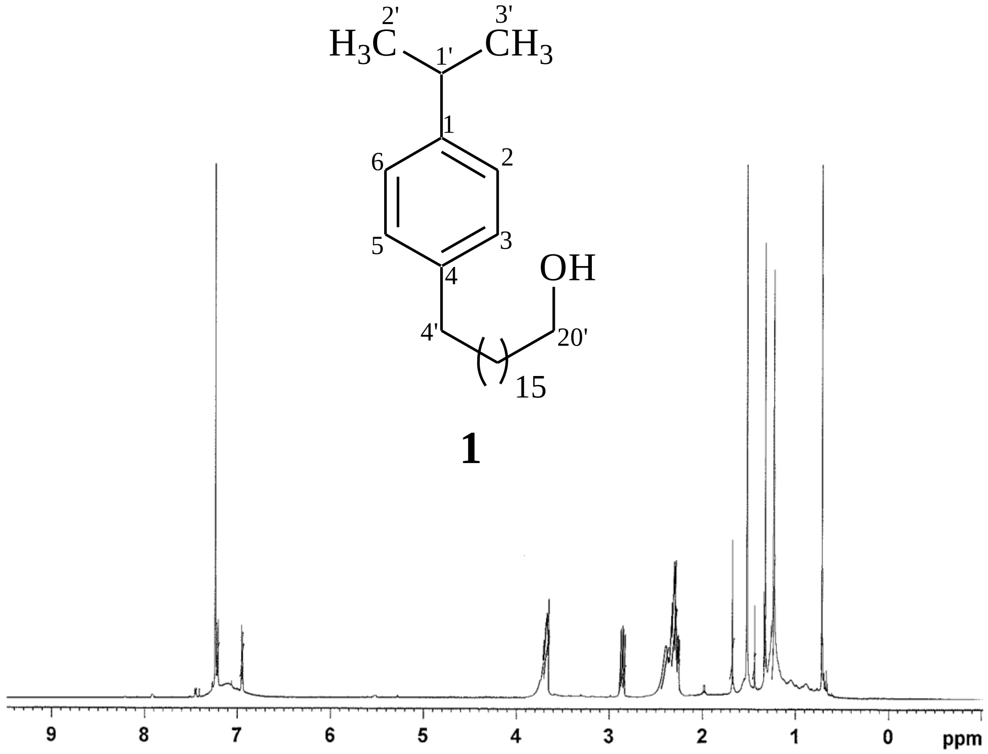

^{13}C NMR Spectrum of Compound **1**

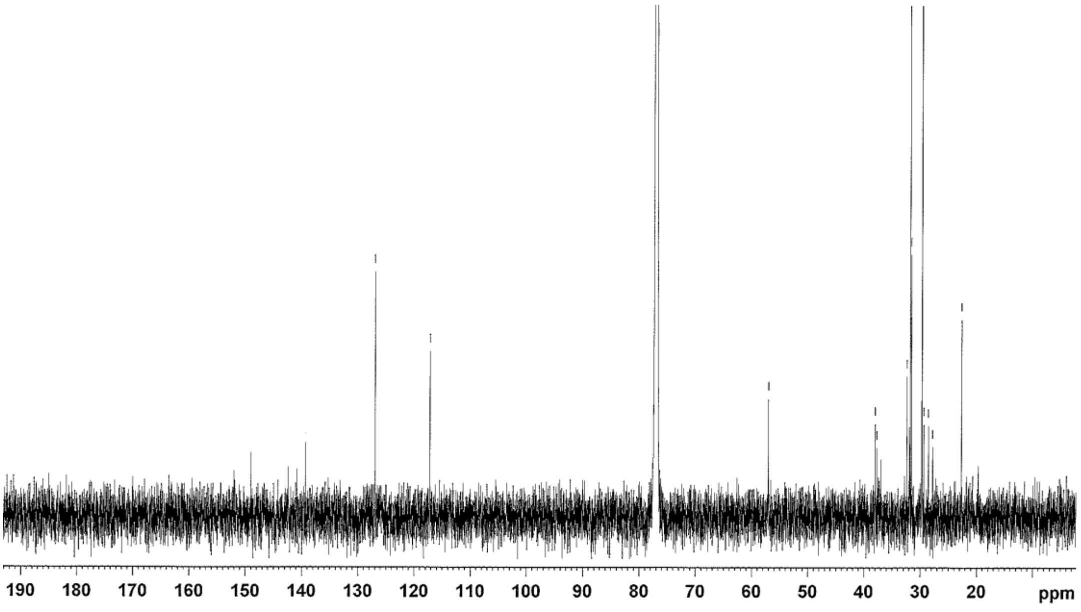

^{1}H NMR Spectrum of Compound **2**

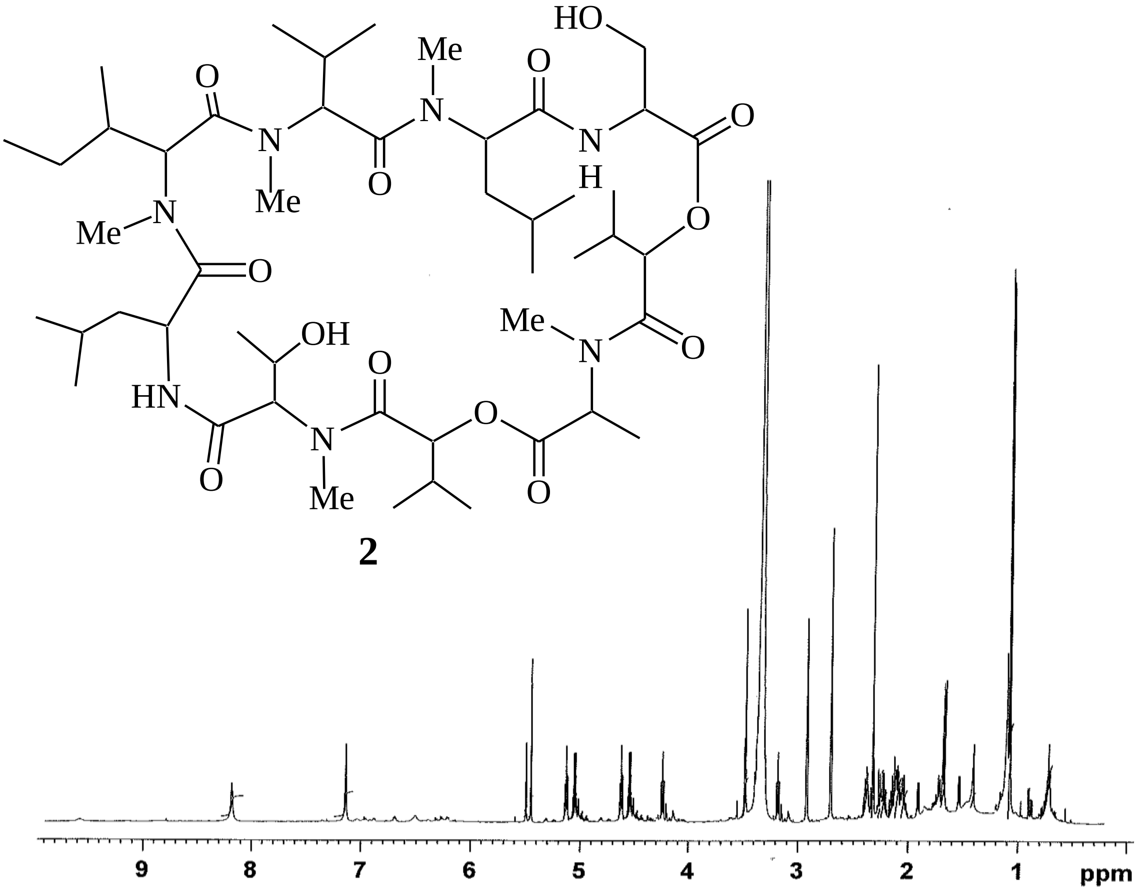

2

^{13}C NMR Spectrum of Compound **2**

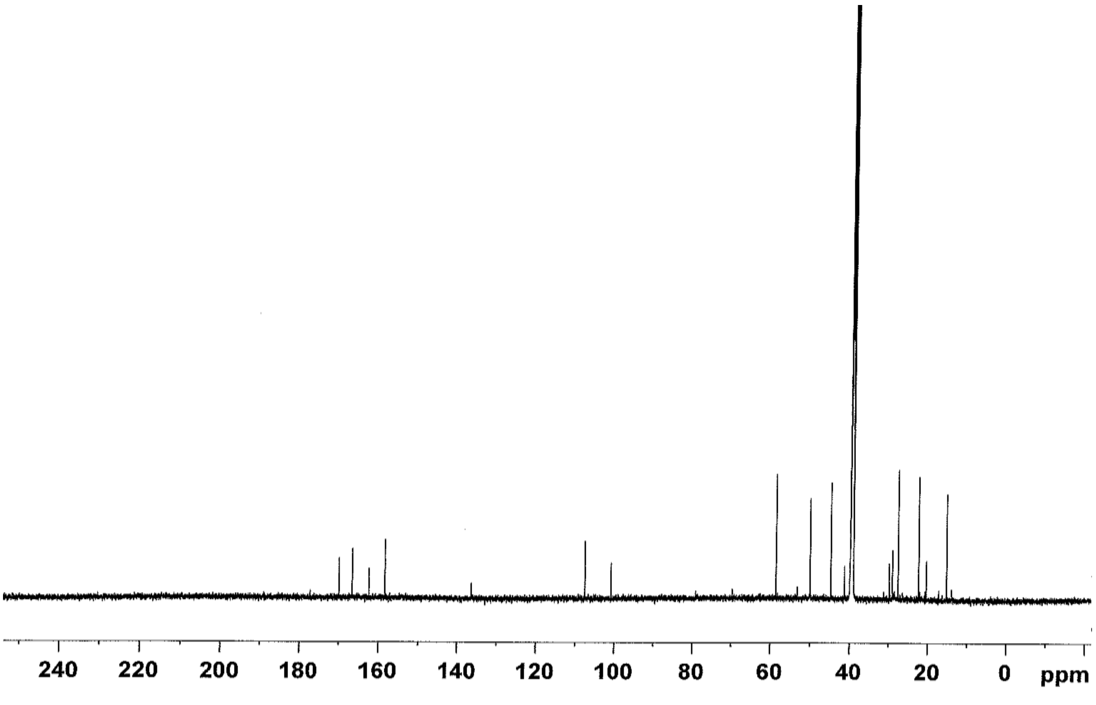

^{1}H NMR Spectrum of Compound **3**

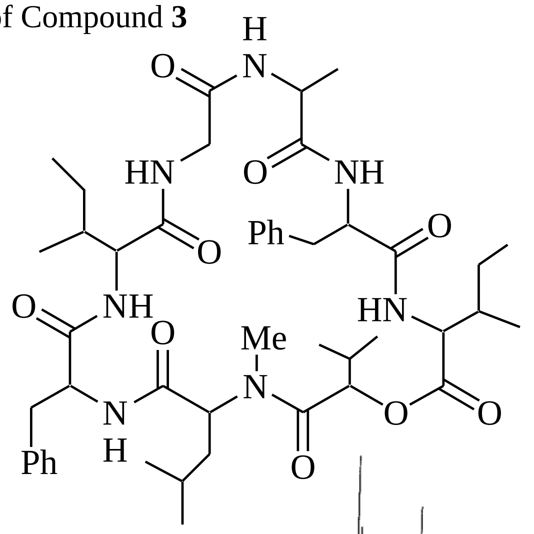

3

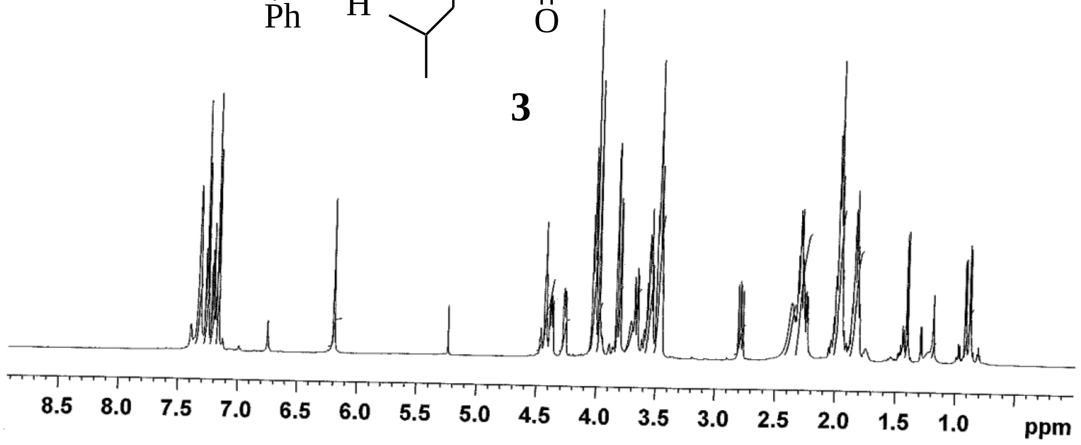

^{13}C NMR Spectrum of Compound **3**

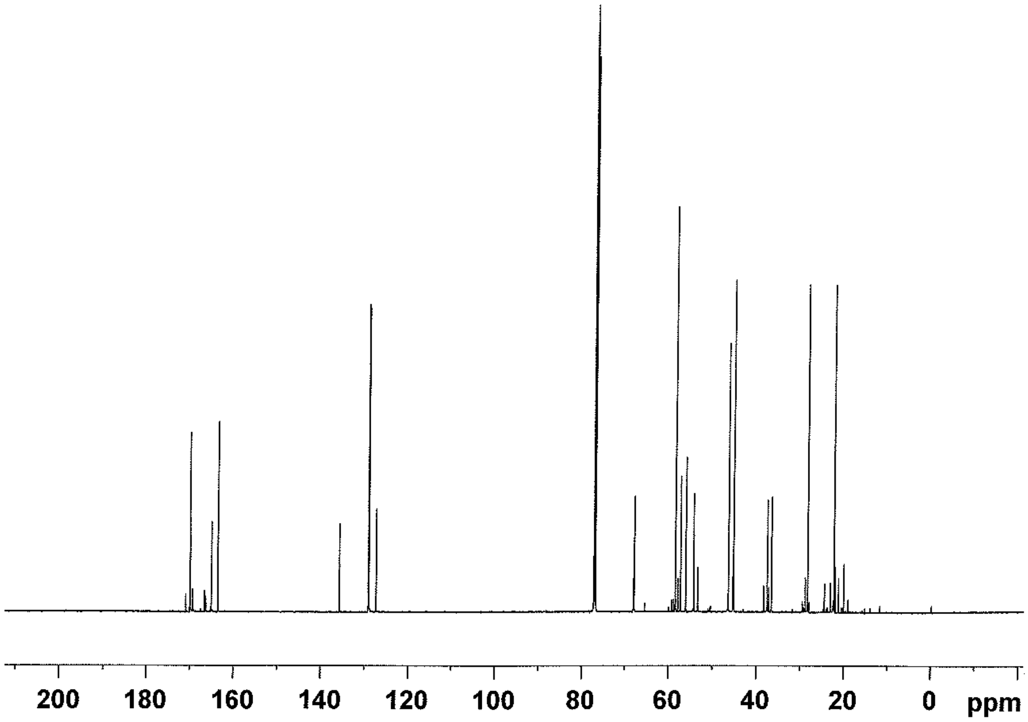

Supplement: Additional file 2: Figure S2. — Nuclear Magnetic Resonance spectroscopic analysis of compound 1-3. (PDF 333 kb) [file 12866_2016_722_MOESM2_ESM.pdf]
